# Modulators of Hypothalamic–Pituitary–Gonadal Axis for the Control of Spermatogenesis and Sperm Quality in Vertebrates

**DOI:** 10.3389/fendo.2014.00135

**Published:** 2014-08-18

**Authors:** Rosaria Meccariello, Silvia Fasano, Riccardo Pierantoni, Gilda Cobellis

**Affiliations:** ^1^Dipartimento di Scienze Motorie e del Benessere (DiSMEB), Parthenope University of Naples, Naples, Italy; ^2^Department of Experimental Medicine, Second University of Naples, Naples, Italy

**Keywords:** spermatogenesis, sperm quality, vertebrates, hypothalamic–pituitary–gonadal, HPG-axis

In both male and female, gametogenesis is regulated by hypothalamus–pituitary–gonadal axis (HPG) that corresponds to the hormonal axis, gonadotropin-releasing hormone (GnRH)–gonadotropins–steroids. Indeed, the main target of GnRH is the gonadotrope cells, located in the adenohypophysis. These, in turn, release two gonadotropin hormones, the follicle stimulating hormone (FSH) and the luteinizing hormone (LH), that through the main circulation reach gonads to regulate gametogenesis via the synthesis of steroid hormones. It is now accepted that further non-steroid factors support germ cell progression via intragonadal action (1).

The first evidence of relationships between pituitary and gonad came out in 1905 from a study on castrated animals, which showed hypertrophy of the pituitary gland (2). Later in 1910, Homans and co-workers (3) showed that the “experimental hypophysectomy” in prepubertal animals induced persistence of gonadal infantilism. Surprisingly, only in 1930 the reciprocal relationship between gonads and pituitary via feedbacks was elucidated (4). Later in 1954, the long feedback connecting the hypothalamus and the gonad was described (5), but only in the 1970s did the picture become complete through the description of the short- and ultrashort-feedback mechanisms. It was at the end of the 1970s that paracrine and autocrine communications were described as being carried out also by “classic” hormones (6). In particular, it was observed that chemical messengers acting through the bloodstream could be produced in multiple tissues, not necessarily including any of the traditional ductless glands. This observation led to the new definition of what constitutes a hormone by considering its function (óρμáω, to excite) rather than its source (ductless glands). A hormone may now be considered as a chemical messenger acting through endocrine (bloodstream), paracrine, and/or autocrine (local) routes. Furthermore, any chemical mediators, not only hormones, besides the endocrine route may also act locally in the gonad (7, 8).

In the testis, it has been demonstrated that a network of intragonadal endocrine, paracrine, and autocrine factors converge in a complex stage-specific multi-factorial control of spermatogenesis (6). Indeed, it has been documented that traditional endocrine control does not fully account for testis physiology, including steroidogenesis and spermatogenesis, and an intragonadal network of autocrine and/or paracrine regulators also exist, which regulates germ cell progression and development of qualitatively mature spermatozoa via cell-to-cell communication (9, 10).

The aim of this Research Topic is to give a comparative track on HPG axis activity for the control of spermatogenesis and quality sperm production. Through synergy between the respective specializations of all the authors, this Research Topic reviews the emerging knowledge about neuroendocrine and local mediators controlling progression and maturation of germ cells in male vertebrates.

The Research Topic firstly reports the description of a primitive HPG in hagfish, one of the only two extant members of the class of agnathans – the most primitive vertebrates known, living or extinct – providing evidence that there are neuroendocrine–pituitary hormones that share common structure and functional features compared to later evolved vertebrates (11). A complex set of neuronal network converges information concerning environmental, stressors, and metabolic cues onto the centers governing the reproductive axis. In this respect, the most recent discoveries in the central pathways integrating metabolism and reproduction in teleost fish have been reviewed here (12). However, the list of central and local modulators of HPG is growing up and currently comprises gonadotropin-inhibiting hormone, firstly identified in Japanese quail in 2000 (13) as an inhibitor of gonadotropin synthesis and release but subsequently identified in all vertebrates (14); classical female hormone such as estrogens that elicit their activity through genomic and non-genomic mechanisms (15); lastly endocannabinoids (16), a set of lipid mediators that share some of the effects with delta-9-tetrahydrocannabinol (Δ^9^-THC), the active principle of marijuana plant, *Cannabis sativa*. The middle part of this Research Topic comprises a set of four review articles dedicated to the control of fetal and postnatal development of both Leydig and germ cells and to the intragonadal networks controlling the progression of the spermatogenesis (17–20); two original research articles point out the discussed involvement of new players such as kisspeptins in the local control of testis physiology (21) and the difficulties to reproduce the testicular environment *in vitro* to get a successful spermatogenesis (22). Lastly, in order to gain the production of high quality sperm, the importance of antioxidant defenses (23), GnRH, kisspeptins, estradiol (24), and endocannabinoids (25) has been reported.

The last part of this Research Topic is focused on disease models such as Kallmann Syndrome (26), blindness (27), lysosomal storage disease (28), and cryptorchidism (29).

We hope that this contribution published in Frontiers in Endocrinology may represent a comprehensive guide in the plethora of data concerning the control of male reproductive activity and that readers might find new insights for the building of general models.

## Conflict of Interest Statement

The authors declare that the research was conducted in the absence of any commercial or financial relationships that could be construed as a potential conflict of interest.
